# Health Professional Students Prefer Study Advice from Institutionally Affiliated Sources Over Internet “Med-fluencers”

**DOI:** 10.1007/s40670-025-02447-z

**Published:** 2025-06-19

**Authors:** Andrew S. Cale, Margaret A. McNulty

**Affiliations:** Department of Anatomy, Cell Biology, & Physiology, Indiana University School of Medicine, 635 Barnhill Drive MS-5035, Indianapolis, IN USA

**Keywords:** Gross anatomy, Trustworthiness, Near-peer, Study strategies, Med-fluencer

## Abstract

Students have access to countless sources of study advice, including medical professionals on social media known as “med-fluencers” However, not all sources are credible. This study explored who students viewed as credible sources and identified any factors that influenced their credibility. Prior to the first exam, allied health students in a doctoral-level gross anatomy course were presented with study advice videos featuring either a “med-fluencer,” an anatomy professor from an external institution, an anatomy professor from the home institution, and a near-peer student. Students then completed the Measure of Ethos/Credibility (MEC), a construct that assesses the perceived competence, goodwill, and trustworthiness of an instructor. After the exam, students completed the MEC again. Students who completed both MECs were then invited to participate in a focus group. A total of 35 students completed the pre-exam MEC, and 16 students completed the post-exam MEC, resulting in 10 matched pairs. Before and after the first exam, the home professor received the highest MEC score, followed closely by the external professor and near-peer student, then the med-fluencer. Thematic analysis also indicated that although the near-peer had the least expertise, students preferred their advice due to their recent experience in their specific course. Overall, allied health students viewed professors as the most credible sources of study advice but preferred to use the advice of near-peers due to their recent experience in their specific gross anatomy course. Conversely, the med-fluencer was viewed as the least credible due to their distance from the students and their course.

## Introduction

In the digital age, students have access to a near limitless number of resources for learning the medical sciences. Social media is one such digital resource that has increased in both popularity and usage amongst medical professionals, educators, and the public in recent years. By 2015, over 90% of 1004 medical students and 4033 clinicians surveyed already reported using social media, with 65% of those clinicians using social media for academic or professional purposes [1, 2]. As a result of the COVID-19 pandemic and the forced transition to digital learning, social media usage has increased even further, becoming near ubiquitous [3]. In 2020, a search for the hashtags “medicine” and “doctor” on TikTok returned 1.4 billion and 6.7 billion views, respectively, with video topics ranging from guidance on applying to medical school, rapid dissemination of information, providing a sense of community, and educating the public [4]. The hashtag “MedEd” was also viewed 4.6 million times on TikTok [4]. However, as in the context of medicine, social media can be a double-edged sword with significant advantages and disadvantages in medical education as well.

According to a 2020 scoping review performed by Chan et al., the number of studies focusing on social media in medical education has risen dramatically since 2005, resulting in over 600 studies spanning a plethora of social media platforms and learner populations [5]. Facebook has been used to create online communities for medical students to share study material, discuss clinical cases, organize face-to-face sessions, and manage medical school-related stress [6, 7]. Online journal clubs and live chats facilitated on Twitter/X, a microblogging platform, have also allowed medical professionals, educators, and trainees to showcase innovative research or pedagogy, practice critical thinking, stay updated with the current literature, and network with the larger community [8–10]. Instagram, a photography-based platform, has also been leveraged for the education of inherently visual disciplines such as histology and radiology [11, 12]. Additionally, a plethora of studies have used YouTube, a video-sharing platform, to share educational or instructional videos focusing on a variety of topics ranging from anatomical knowledge to clinical techniques [13]. Wikis, collaboratively written and edited web pages, built around specific medical specialties, were also viewed as useful resources for reviewing clinical material, but did not significantly impact trainee knowledge [14]. These examples represent a small fraction of the many applications of social media, though their long-term educational impacts require further exploration, especially as the social media landscape and their capabilities continue to evolve.

As both the public and medical professionals increasingly turn to social media, public figures known as med-fluencers (a portmanteau of the words *medical* and *influencer*) have gained prominence in the public consciousness [15]. These individuals use their social media clout to influence the opinions and behaviors of their audiences on topics related to health and medicine. However, despite their positions of social authority, med-fluencers may or may not have the appropriate qualifications to speak on or advise their audiences on issues related to health, science, or education [16]. As a result, highly qualified and experienced medical professionals, scientists, or educators may be held in the same regard as non-experts who knowingly or unknowingly disseminate misinformation.

Like their non-medical social media influencer counterparts, med-fluencers participate in brand collaborations in which they advertise or endorse a brand’s product in exchange for financial compensation [17]. For example, the marketing agency, Medfluencers™, leverages the social media clout and reach of their medical professionals for the specific purpose of promoting products [18]. In the context of medical education, these promotions may involve textbooks, digital applications, tutoring/test-prep services, and other educational resources. Given their professional authority and social influence, students may find it challenging to assess the credibility of med-fluencers and the usefulness of their promoted study strategies and resources.

The purpose of this study is to explore how students perceive sources of study advice, specifically med-fluencers, professors, and near-peers, using McCroskey’s source credibility framework [19]. In this model, credibility includes three domains: (1) competence—subject matter knowledge and intelligence, (2) goodwill—positive intent, and (3) trustworthiness—honesty and character [19, 20]. These domains collectively influence whether students consider a source credible, affecting their likelihood of adopting recommended learning strategies and ultimately shaping their learning outcomes. A multiple-methods approach, incorporating both quantitative questionnaires and qualitative focus groups, addressed the following research questions: (1) Who do students perceive as credible sources of study advice? (2) What factors influence these perceptions? The insights gained from this study will help educators determine which factors shape a student’s perception of credibility, allowing them to offer study advice in ways that resonate better with students.

## Methods

### Study Overview and Participant Recruitment

This study was given exempt status by the Indiana University Institutional Review Board (Protocol #15,123). During the summers of 2022 and 2023, 240 first-year students from the Indiana University School of Health and Human Sciences (*n*_2022_ = 120, *n*_2023_ = 120) were invited to participate in this study, including 84 Doctor of Physical Therapy (DPT; *n*_2022_ = 41, *n*_2023_ = 43), 88 Master of Physician Assistant Studies (MPAS; *n*_2022_ = 44, *n*_2023_ = 44), and 68 Doctor of Occupational Therapy (OTD; *n*_2022_ = 35, *n*_2022_ = 33) students.

### Course Description: Anatomy for Healthcare Professionals

#### Course Overview

As part of their first-year curricula, students were enrolled in “Anatomy for Healthcare Professionals.” This five-credit hour, doctoral-level interprofessional gross anatomy course was designed to introduce students to the basic concepts and structures of the human body. Students progressed through the anatomy of the human body using a dissection-based curriculum. Lectures were pre-recorded and delivered asynchronously through the institution’s Canvas LMS learning management system (Thoma Bravo LLC., Chicago, IL). Summative assessments were administered following the completion of each of the four course blocks, which included both lecture and laboratory (practical) components.

#### Study Strategy Videos

One week prior to the first block exam, students were given access to a Canvas page with four short videos that described effective study strategies for learning gross anatomy. Each video featured a different speaker: a popular internet “med-fluencer,” an anatomy professor from an external institution, an anatomy professor from the home institution, and a near-peer graduate student from the home institution. These individuals were chosen because they represented a spectrum in social media presence and proximity to the students, with the med-fluencer being the most prominent social media figure with the least relation to the students and the near-peer being the least prominent but most related to the students. All speakers presented as themselves except for the near-peer student, who went by a pseudonym. More details on the speakers’ personal and video characteristics can be found in Table 1. In each video, the speaker described a standardized set of study strategies in their own words and style: (1) study consistently to keep up with the material, (2) practice active recall, (3) make connections across the material, (4) study purposefully, and (5) be open to trying new study strategies. These specific study strategies were distilled through content analysis of over 100 videos from a previous cohort of allied health students in which they detailed their advice for future students. To maintain authenticity, each speaker was allowed to present the standardized study strategies in their own words. However, other aspects of the delivery were standardized, with each video recorded in the style of an informal video message or conversation (e.g., Zoom meeting or FaceTime call) with no editing or added production value from recording equipment, lighting, scripting, set design, props, costumes, hair and make-up, on-screen text, and digital or practical effects. The length of the videos did differ slightly due to the tone, speed, and mannerisms of each speaker, but were still similar in length, ranging from 2:20 to 3:33 min. As such, all four videos were comparable in both content and delivery.

**Table 1:** Personal and video characteristics of speakers

	**Video speaker**			
	**Med-fluencer**	**External faculty**	**Internal faculty**	**Near-peer student**
Personal characteristics				
Age range (years old)	40–50	40–50	40–50	20–30
Race/ethnicity	White	White	White	White
Gender	Male	Male	Male	Female
Profession	Physician	Professor	Professor	Graduate student
Social media presence	Large (~ 1.6 million followers)	Large (~ 300,000 followers)	Medium (~ 2400 followers)	None
Video characteristics				
Length (min)	3:33	3:17	2:20	2:43
Recording device	Smartphone	Webcam (desktop or laptop)	Webcam (desktop or laptop)	Webcam (desktop or laptop)
Video editing	None	None	None	None

### Data Collection

#### Questionnaires

Before and after the first exam, students were invited to complete a questionnaire containing the Measure of Ethos/Credibility (MEC) for each of the four video speakers. Developed by McCroskey and Teven, the MEC is an 18-item survey instrument that assesses an individual’s perceived credibility [19]. The MEC has been implemented in a variety of contexts, including to capture the perceived credibility of social media influencers, educators, AI-generated instructors, and product reviewers [21–26]. As such, the MEC is the ideal instrument for capturing student perceptions of the med-fluencer, professors, and near-peer in the current study.

In alignment with McCroskey’s source credibility framework, the MEC assesses credibility across three domains: (1) competence, (2) goodwill, and (3) trustworthiness [19]. Each domain includes six items on a seven-point semantic differential scale (e.g., 1 = inexpert, 7 = expert), with some items reverse-coded to encourage participants to focus and avoid straight line survey responses (i.e., one response value across all survey items) [19]. A domain score is calculated by summing the responses within the domain of competence, goodwill, or trustworthiness (after reverse-coding as appropriate), resulting in a score between 6 (minimum) and 42 (maximum) [20]. A total MEC score is then calculated by summing the three domain scores, resulting in a score between 18 (minimum) and 126 (maximum) [20]. Higher scores indicate greater perceived competence, goodwill, trustworthiness, or overall credibility by the student.

In accordance with the argument-based evidentiary chain model, the use of the MEC instrument under these conditions was supported through re-evaluation of existing validity evidence and gathering of new evidence [27]. Given the similarity of the current study’s appraisers (students), appraisees (educators and social media influencers), and overall context to previous studies, complete re-validation of the MEC instrument was considered unnecessary [21–25]. In these previous studies, the overall MEC instrument demonstrated high internal consistency (*α* = 0.90–0.94), as did the individual domains of competence (*α* = 0.84–0.92), goodwill (*α* = 0.82–0.92), and trustworthiness (*α* = 0.86–0.92) [19, 21–26]. In the current study, the overall MEC exhibited an internal consistency of 0.96, whereas the individual domains of competence, goodwill, and trustworthiness exhibited internal consistencies of 0.93, 0.92, and 0.88, respectively. Experts in medical education also reviewed the content validity of the MEC under the current study conditions and deemed it appropriate. Lastly, the response processes for the MEC were evaluated using a think-aloud protocol, where students verbalized their thoughts while completing the instrument to confirm they understood and answered the items correctly.

#### Post-Exam Focus Group

After the first exam, students who completed both the pre- and post-exam trustworthiness questionnaires were invited to participate in a 30-min focus group session via Zoom (Zoom Video Communications, Inc., San Jose, CA). These focus groups included between two to four students and followed a semi-structured format and included ten questions that were always asked of the participants (Appendix). These questions pertained to the students’ impressions of the speakers and any factors that may have influenced those impressions such as speaking tone, professions, age, gender, social media presence, and any other factors that were considered relevant. Otherwise, the conversation was allowed to expand or deviate on topics that were not initially part of the question list but considered relevant to the students’ perceptions of the video speakers. To ensure accurate capture of the discussion, the focus group sessions were recorded, and all dialogue was transcribed verbatim using Otter.ai (Otter.ai, Inc., Mountain View, CA).

### Statistical Analysis

All quantitative data were exported from their original platforms and imported into SPSS analysis software, version 29 (IBM Corp., Armonk, NY) for analysis. Descriptive statistics were calculated for the Competence, Goodwill, and Trustworthiness subdomains for each speaker, and data were visually tested for normality using histograms. Due to the non-normal nature of the data, non-parametric methods of inferential statistics were applied. The Kruskal–Wallis *H* test was used to compare subdomain scores across the four speakers at the pre- or post-exam timepoint. The related-samples Wilcoxon signed rank test was also used to compare the pre- and post-exam subdomain scores for a single speaker. Results for all statistical analyses were considered significant if *p* < 0.05.

### Qualitative Analysis

Focus group transcripts were deidentified and imported into Dedoose qualitative analysis software (version 9.0.62, SocioCultural Research Consultants, LLC, Los Angeles, CA) for thematic analysis. An inductive thematic analysis following the method described by Braun and Clarke was performed, allowing the data to drive code and theme generation [28]. First, one researcher (A.S.C.) reviewed all focus group transcripts to develop familiarity with any items of interest. A codebook derived from the data was then generated and applied to all focus group transcripts. After the coding process, codes were organized into provisional themes based on their relationships to one another. Lastly, the provisional themes were reviewed, defined, and named.

## Results

A total of 35 students opted to participate in this research study, including 17 DPT, 11 MPAS, and 6 OTD students. Participant demographics were comparable between the 2022 and 2023 cohorts (Table 2).

**Table 2:** Participant demographics

	**2022**	**2023**	**Total**
Self-reported gender			
Female	14	13	27
Male	5	2	7
Non-binary	0	0	0
Declined to answer	0	1	1
Program of study			
Doctor of Physical Therapy (DPT)	9 (22.0%)	8 (18.6%)	17 (20.2%)
Master of Physician Assistant (MPAS)	7 (15.9%)	5 (11.4%)	12 (13.6%)
Doctor of Occupational Therapy (OTD)	3 (8.6%)	3 (9.1%)	6 (8.8%)

Each percentage reflects the portion of participating students out of the eligible students in that group.

### Study Strategy Video Data

Viewership of the study strategy videos was similar between 2022 and 2023. In 2022, the med-fluencer’s video was viewed the most (*n* = 47 views), followed by the external faculty (*n* = 39 views), the internal faculty (*n* = 38 views), and the near-peer student (*n* = 36 views). In 2023, the med-fluencer’s video was viewed the most once again (*n* = 48 views), followed by both the external faculty and the near-peer student (41 views each), and then the internal faculty (*n* = 33 views). Due to the limitations of viewer data available through the video platform at the time, it is not possible to determine if these views are from unique or repeat viewers.

### Measure of Ethos and Credibility (MEC)

**Fig. 1:**
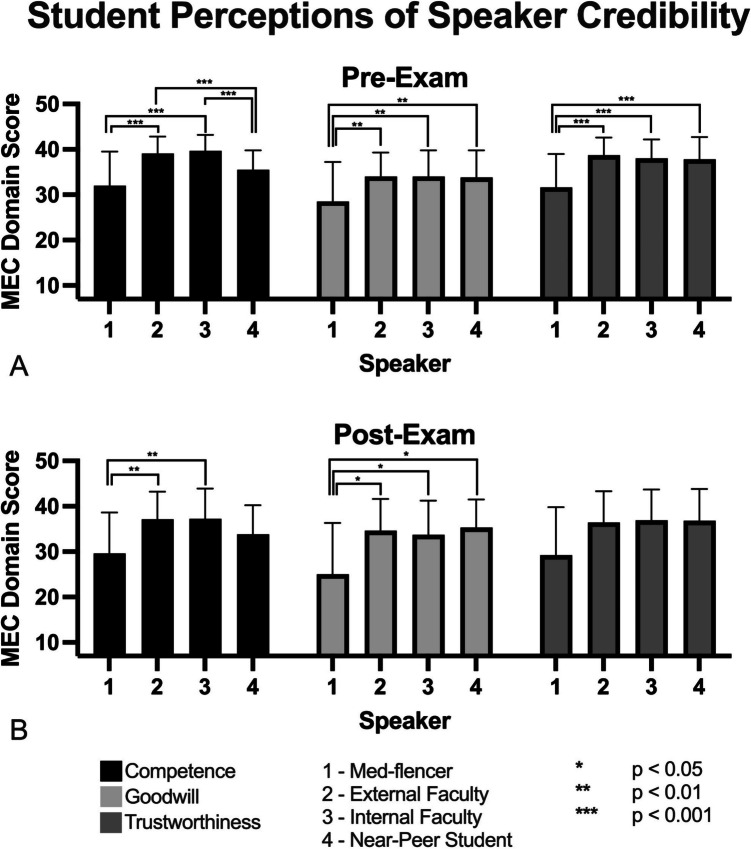
Student perceptions of speaker credibility. **A** Pre-exam student perceptions of speaker competence, goodwill, and trustworthiness by speaker. **B** Post-exam student perceptions of speaker competence, goodwill, and trustworthiness by speaker. Measure of Ethos and Credibility (MEC) includes three domains (Competence, Goodwill, and Trustworthiness), each with a minimum and maximum possible scores of 7 and 42, respectively

Between 2022 and 2023, 35 students (*n*_2022_ = 19, *n*_2023_ = 16) completed the pre-exam MEC whereas 16 (*n*_2022_ = 8, *n*_2023_ = 8) completed the post-exam MEC, resulting in 10 matched pairs (*n*_2022_ = 6, *n*_2023_ = 4). Prior to the first block exam, survey participants viewed the external and internal faculty members as comparably competent in anatomy (*p* > 0.05), but significantly more competent than both the med-fluencer and the near-peer graduate student (*p* < 0.001). The external faculty, internal faculty, and near-peer graduate student were also perceived as similarly goodwilled and trustworthy to one another (*p* > 0.05), though all three were considered significantly more goodwilled and trustworthy than the med-fluencer (*p*_goodwill_ < 0.01; p_trustworthy_ < 0.001). After the first block exam, the external and internal faculty members were still considered significantly more competent than the med-fluencer (*p* < 0.01). However, they were no longer viewed as significantly more competent than the near-peer graduate student (*p* > 0.07). The external faculty, internal faculty, and near-peer graduate student also continued to be viewed as significantly more goodwilled than the med-fluencer (*p* < 0.02). Lastly, all four speakers were now perceived as similarly trustworthy to one another (*p* > 0.05) (Fig. 1). When pre- and post-exam MEC scores were individually matched to survey participants and compared, no significant changes in MEC scores were identified for any of the speakers.

### Focus Group Sessions

Several themes were identified through inductive thematic analysis of the focus group transcripts, including: *Home Advantage*, “*Expert Enough*,”* Irrelevance of Social Media*, and *Awareness of Insincerity*. Quotes have been edited for grammar, clarity, conciseness, and to maintain anonymity.

#### Home Advantage

Focus group students considered the study advice from sources closely associated with their specific university, program, and course, such as the internal faculty and near-peer graduate student, as more trustworthy due to variations in how the same material could be taught in different contexts. Conversely, the study advice from individuals who were more distanced from the students was viewed as less relevant or applicable:

“*Since* [the near-peer student] *took this class, I feel like she knew the best about what we were going through just because I mean taking anatomy can differ between which university you’re at and what program you’re in…I felt like I could trust her knowing that she took the exact same class and did well in it*.”

“*Just because* [the internal professor’s] *advice is more relative to what we would have to know since there’s variations in what’s taught and what the professor from* [the external institution] *goes into*.”

#### “Expert Enough”

Although the focus group participants perceived the near-peer graduate student as less knowledgeable in gross anatomy, they considered her to be “expert enough” to provide effective study advice. Her advice was viewed as more relevant due to her recent successful experience in the same course the students are enrolled in. Conversely, participants believed faculty members were sometimes too distanced from their current situation to provide relevant advice:

“*I almost thought* [the near-peer student’s] *advice was the best when it came to studying because she’s the one who has done it the most recently. The only thing I thought about her was like her expertise as far as an anatomy in general was probably not as high as the others because they are professionals in their field where she was just a student, but I think that her advice for studying was the most relevant for me.*”

“*Sometimes somebody that’s more at the same level or just like a little bit farther along than you are can give advice that’s very applicable to where you’re at right now, whereas somebody that’s much much farther advanced in their career doesn’t* [give] *advice that feels as applicable to what we’re doing right now.*”

#### Awareness of Insincerity

The focus group participants were also closely attuned to the sincerity of the speaker in each study strategy video. Despite the standardized content, students found the med-fluencer more self-centered and noted that his delivery felt scripted and aloof. This perceived insincerity caused the students to mistrust the med-fluencer’s study advice:

“*I thought that the* [the med-fluencer] *was also just kind of a one off in the hallway. He didn*’*t put much effort into it. Like* [the course director] *just asked him to make the video on the fly versus it looking like some of the other people who put a little bit more effort into where and when they were. It’s the thoughtfulness they put into the video. So that kind of made a difference to me*.”

“[The med-fluencer] *doesn’t have as much connection with our program. A lot of the video was spent talking about himself versus us. So, I feel like that ranks the lowest for me.*”

Conversely, students preferred the more caring tone and calming presence of the other speakers because it made them feel like the speaker was invested in their success.

“*I feel like* [the internal faculty] *was just so smooth, calming, and family sounding. I don’t know, it was like, “Okay, I got it. I can do it.” I don’t need someone to someone to make things too energetic. I like them calming down things because there’s nothing calm in the anatomy class right now.*”

#### Novelty of Social Media

Several participants also noted the social media presence (or lack thereof) of the speakers’ but did not believe it directly influenced their judgments of the speakers’ trustworthiness. Rather, the large social media followings were treated more as a novelty that piqued the participants’ interest and encouraged them to view the speakers’ profiles and content. Participants also perceived certain social media platforms as more for entertainment (e.g., TikTok) whereas others were more for educational purposes (e.g., YouTube).

“*I think* [the med-fluencer] *has like over a million or some really, really high TikTok following or something…But I don’t think that it made me think that he’s going to be more knowledgeable than the next because I see a lot of people that have massive following* [but] *it’s very dumb content*.”

“*I remember looking at the follower numbers on the* [Canvas] *page, but I didn’t really take it into account either. Thinking about social media,* [the med-fluencer] *is just on TikTok more for entertainment. And so that’s how his video was directed, whereas* [the external faculty’s] *huge YouTube following is based on needing to know information and learning. I think he’s knowledgeable just as much as* [the internal professor] *is. It’s just that* [the internal professor] *doesn’t choose to record extra videos in his free time to post on YouTube*.”

## Discussion

In this study, doctoral-level allied health students perceived the speakers of study advice videos differently despite standardization of content across the videos. Both the external and internal faculty members were consistently perceived as the most competent, goodwilled, and trustworthy sources of anatomy study advice prior to and after the first exam. Initially, the near-peer graduate student was considered similarly goodwilled and trustworthy in comparison to the faculty, but significantly less competent in anatomy. However, after the first exam, the near-peer graduate student’s perceived competence in anatomy was not significantly different from that of the faculty. Lastly, the med-fluencer was rated the lowest in terms of competence, goodwill, and trustworthiness when giving anatomy study advice both before and after the first exam. Focus group interviews provided further insight into these student perceptions of the four video speakers. Students preferred study advice from sources closely associated with their specific course, program, or university because their advice was viewed as more relevant. Similarly, students valued the study advice from near-peers because of their recent successful experience in the same course, making their advice more relevant and practical. Also, when searching for study advice, students are closely attuned to the sincerity of the source of the study advice, preferring those with a caring and calming tone over those that are more aloof. Students also viewed study advice from med-fluencers as novel and entertaining, but do not believe a speaker’s social media presence influences their judgments of study advice quality. As such, these allied health students are more discerning with who they trust for advice, taking into account several critical factors that other social media users may not consider. These insights into who students view as credible sources of study advice and why can help medical educators provide students with study advice in a more effective manner.

### Perception of Professors

As demonstrated by both the MEC and focus group sessions, students perceived faculty as the best resources for both content and study advice, regardless of their institutional affiliation. In both the pre- and post-exam MECs, the external and internal professors were consistently rated as the most competent, good-willed, and trustworthy speakers. Similarly, in the focus groups, students viewed the professors as “professionals in their fields” who provided them with calm reassurance of their learning. This view of professors as supportive pedagogical experts is partially consistent with the existing literature. On the positive side, professors are generally perceived as knowledgeable, experienced, respected, confident, in control, and organized [29]. Roughly 78% of the public also believes that professors have a positive influence on society [30]. More specifically, in the eyes of undergraduate and graduate students, professors are valued for their pedagogical knowledge, such as general and content-specific teaching methodologies, more so than even their content knowledge [31]. However, professors are also sometimes seen as distant, formal, strict, and boring [29]. This colder view contrasts with how students perceived the professors in this study and may be related to their limited exposure to or the qualities of the speakers portrayed in the videos. According to Nushi et al., a student’s assessment of a professor is based on multiple factors, particularly assessment policies and practices, personality, and pedagogical knowledge [31]. In the case of the study advice videos, students were presented with a brief, highly curated portrayal of the professors, which may have positively biased their opinions. Together, these factors may explain the students’ highly positive perception of the professors in both the MEC and focus groups.

Although students viewed them as comparable, they also preferred study advice from professors at their own institution when given the option due to proximity to the course. This preference for resources from affiliated sources likely originates from the belief that exam questions will be closely aligned with course materials. As the focus group students noted, “*anatomy can differ between which university you’re at and what program you’re in*” and the internal professor’s advice is “*more relative to what we would have to know since there’s variations in what’s taught.*” Based on previous studies, course-specific materials, particularly lectures and lecture notes, were consistently reported by medical students as their preferred and most regularly used resources for self-directed study and learning new material [32, 33]. Post-pandemic, 94.7% of 379 surveyed medical students continued to report instructor-generated lecture slides as their primary study resource [34]. The majority of medical students believed that more than 80% of exam questions would be drawn from the lecture material, which explains the preference for course-affiliated materials or advice [34]. Similar material may be presented across anatomy courses, but the specificity or assessment method by which students may be tested on a concept may differ from course to course. Therefore, it is reasonable for students to prefer resources or advice that will be closely aligned with their assessments, as was the case in this study.

While professors were viewed as both content and pedagogical experts, students believed they sometimes provided advice that was not immediately applicable to their current learning needs. For example, one focus group participant stated that “*somebody that’s much much farther advanced in their career doesn’t [give] advice that feels as applicable to what we’re doing right now.*” This disconnect may stem from the “curse of knowledge,” a cognitive bias where an expert unintentionally assumes a novice learner is more well-versed on a topic than in reality [35]. Since professors are so well-versed in the complexities of a particular topic, some may struggle to simplify their discussions of the topic to the appropriate level of a novice since it has become second nature to them. This “academese” is often filled with language like jargon, abbreviations, technical terms, Latin phrases, or other unnecessary jargon [36]. As such, students may prefer explanations from fellow peers that match their current level of understanding.

### Perception of Near-Peers

Although considered less knowledgeable in content, near-peer students were highly valued as well-meaning sources of relevant and practical study advice, with some students even preferring their advice over advice from the professors. In both the pre- and post-exam MECs, students rated the near-peer student similar to the external and internal professors in terms of competence and goodwill. In the focus groups, students explained that they appreciated the near-peer’s recent experience in their specific course and felt that their study advice was more relevant and applicable. This attitude is consistent with previous studies involving near-peers. Since near-peers typically share a similar academic or professional role, the hierarchical difference between the students and themselves is much smaller. As such, students often find near-peers more approachable, relatable, understanding, and casual than faculty instructors, and are often more inclined to approach their near-peers several times a week for assistance compared to less than weekly for teaching staff [29]. Their recent experience also allows them to provide valuable “tricks of the trade” that can be readily applied to the students’ current needs such as mnemonics [37]. Students are also more likely to use these study resources recommended by peers than by teaching staff [33]. Previous studies have leveraged these advantages of near-peers to enhance teaching and learning in medical education [37–41]. When implemented as anatomy and physiology tutors for medical students, near-peers reinforced class material, improved understanding of difficult concepts, and encouraged students to monitor their learning, particularly for students who are struggling [40]. In another study, neuroanatomy-focused teaching sessions led by near-peers resulted in an 18% improvement in student confidence and perceived knowledge of concepts discussed during the sessions [41]. When placed in a mentorship role, near-peers have also been found to enhance personal growth, develop professionalism, reduce stress, and ease the transition into medical school for junior colleagues [37].

However, one of the notable drawbacks of near-peers is the reliability of their content knowledge and study advice. As noted by one focus group participant, near-peers do not have the same level of content expertise as faculty. Nor are they pedagogical experts. As a result, some near-peers may not be as prepared or as confident when teaching, leading to errors [40]. Given that students are more inclined to approach near-peers for assistance, incorrect information or ineffective advice can spread rapidly through the student population. To mitigate this risk, near-peers in formal educational roles such as teaching assistants or mentors should be properly trained prior to entering their roles.

### Perception of Med-fluencers

Despite their high popularity online and role as a physician, the med-fluencer was viewed as the least credible source of anatomy study advice due to their distance from the students and their aloof attitude. In the MECs, the med-fluencer was consistently rated as the least competent and goodwilled speaker. Several focus group participants echoed this sentiment by pointing out that the med-fluencer “*didn’t put much effort into it*” and spent much of the video “*talking about himself versus us.*” This negative perception of the med-fluencer may be related to multiple factors. Typically, physicians are viewed as authoritative and credible sources of medical information [42]. However, some are highly specialized and may not be perceived as experts in all aspects of medicine or human anatomy. This may have been the case with the med-fluencer who specializes in a very specific organ. As a result, students may not have viewed the med-fluencer as an expert in other anatomical regions that they were also responsible for learning, resulting in a lower rating. The social media platform on which the med-fluencer is active may have also negatively influenced student perception. Although social media platforms have educational potential, some are better suited to in-depth learning of complex material [4]. The med-fluencer is most active on the video-sharing platform, TikTok, which one student viewed as “more for entertainment,” which is aligned with the public’s view as well [15]. Conversely, long-form video-sharing platforms such as YouTube are “based on needing to know information and learning.” Other social media platforms considered to have high educational value that have been adopted by the medical community include podcasts and (micro)blogs [43]. Since the med-fluencer created their video in the style of a TikTok video, the preconceptions students have about the platform and its content may have influenced their opinion.

### Limitations

One key limitation of this research is the variation between the study strategy videos. Although all four video speakers were asked to provide a standardized set of study strategies, the other features of their videos were left to their discretion to allow the videos to feel authentic. As such, the videos differed slightly in their length, dialogue, backgrounds, tones, and overall styles. These variations may have also positively or negatively influenced the viewer’s perceptions of the speaker.

Another limitation is the low number of participants who completed various elements of the study, such as completing the pre- and post-exam MEC or watching the study strategy videos. Although all students enrolled in the “Anatomy for Healthcare Professionals” course were eligible and invited to participate in this study, only 10 students completed both the pre- and post-exam MECs. Similarly, views of the study strategy videos ranged between 33 and 48 views per video, which was well below the class size. As a result, this study may be vulnerable to self-selection and non-response biases. The perceptions of the individuals that chose to watch the videos and participate in the surveys and focus groups may be over-represented whereas the perceptions of those who did not participate may be underrepresented or overlooked. This low participation rate may have been caused by multiple factors including survey fatigue or time constraints. Students may have also opted to forgo watching the study strategy videos for several reasons, including an existing comfort with studying, time constraints, and poor initial impressions of the video and speaker based on the thumbnail images alone.

## Conclusion

Today, health professional students have access to a staggering number of resources for learning the medical sciences, ranging from their own peers to professors to social media med-fluencers. Faced with these overwhelming options, students must constantly judge for themselves which sources are credible and offer accurate, effective advice that can enhance their learning. Based on the findings of this study, students consider institutionally affiliated sources such as their professors and near-peers as the most credible and relevant sources of study advice. Unaffiliated professors were also considered content and pedagogical experts, but their advice may not be as applicable. Lastly, med-fluencers were perceived as the least credible sources of study advice. The insights gained from this study can guide medical educators and help them present study advice in a manner in which students are most receptive.

## Appendix

### Post-Exam Focus Group Questions

1. What were your initial impressions of Speaker #1? Speaker #2? Speaker #3? Speaker #4?
2. Which speaker(s) did you find most trustworthy for anatomy advice? Why?
3. Which speaker(s) did you find least trustworthy for anatomy advice? Why?
4. Did you incorporate any of the speakers’ advice into how you studied? If so, whose and which ones?
5. What were your impressions of the speakers’ tone?
6. What were your impressions of the speakers’ professions?
7. What were your impressions of the speakers’ ages?
8. What were your impressions of the speakers’ genders?
9. What were your impressions of the speakers’ social media presence?
10. Were there any other characteristics that impacted your view of the speakers’ trustworthiness?
